# Breathing variability during propofol/remifentanil procedural sedation with a single additional dose of midazolam or s-ketamine: a prospective observational study

**DOI:** 10.1007/s10877-021-00773-2

**Published:** 2021-11-12

**Authors:** O. F. C. van den Bosch, R. Alvarez-Jimenez, S. G. Schet, K. Delfsma, S. A. Loer

**Affiliations:** Department of Anesthesiology, Amsterdam UMC, VUMC, De Boelelaan 1117, ZH 6F 003, 1081 HV Amsterdam, The Netherlands

**Keywords:** Respiratory volume monitoring, Procedural sedation, Breathing variability, s-Ketamine, Midazolam

## Abstract

**Purpose:**

Regulation of spontaneous breathing is highly complex and may be influenced by drugs administered during the perioperative period. Because of their different pharmacological properties we hypothesized that midazolam and s-ketamine exert different effects on the variability of minute ventilation (MV), tidal volume (TV) and respiratory rate (RR).

**Methods:**

Patients undergoing procedural sedation (PSA) with propofol and remifentanil received a single dose of midazolam (1–3 mg, n = 10) or s-ketamine (10–25 mg, n = 10). We used non-invasive impedance-based respiratory volume monitoring to record RR as well as changes in TV and MV. Variability of these three parameters was calculated as coefficients of variation.

**Results:**

TV and MV decreased during PSA to a comparable extent in both groups, whereas there was no significant change in RR. In line with our hypothesis we observed marked differences in breathing variability. The variability of MV (– 47.5% ± 24.8%, p = 0.011), TV (– 42.1% ± 30.2%, p = 0.003), and RR (– 28.5% ± 29.3%, p = 0.011) was significantly reduced in patients receiving midazolam. In contrast, variability remained unchanged in patients receiving s-ketamine (MV + 16% ± 45.2%, p = 0.182; TV +12% ± 47.7%, p = 0.390; RR +39% ± 65.2%, p = 0.129). After termination of PSA breathing variables returned to baseline values.

**Conclusions:**

While midazolam reduces respiratory variability in spontaneously breathing patients undergoing procedural sedation, s-ketamine preserves variability suggesting different effects on the regulation of spontaneous breathing.

## Introduction

Regulation of breathing is highly complex aiming to adapt respiratory muscle activity to metabolic demands. The respiratory centers (located at the medulla and pons) receive inputs from not only peripheral and central chemoreceptors, but also from many other receptors like those sensing pain, or reacting to stretch or irritant agents within the lungs [1]. In addition, ventilation is also influenced by voluntary control of breathing. Thus, the resulting breathing pattern shows significant variability which decreases by 28–52% during sleep [2, 3]. Many anesthetics and opioids influence respiratory regulation through various mechanisms [4, 5]. Some anesthetics decrease respiratory drive with effects on the CO_2_-response curve [5]. In contrast, the N-methyl-D-aspartate (NMDA)-receptor antagonist s-ketamine stimulates breathing and attenuates propofol- and opioid-induced hypoventilation [6–8].

Breathing variability is defined as the extent to which respiratory parameters such as respiratory rate (RR), tidal volume (TV) and minute ventilation (MV) fluctuate over time [3]. It has been studied for many years and it has been regarded as an indicator of respiratory function [3, 9–16]. Interestingly, a higher risk of childhood asthma was observed in children with less tidal flow variability [12]. In another study, spontaneously breathing intensive care patients with reduced breathing variability had a higher risk of unsuccessful separation from mechanical ventilation [13]. We recently observed that after major abdominal surgery, variability in RR is smaller than variability in TV and MV; and that variations in RR are independent of changes in TV or MV [17]. It has been suggested that central neural mechanisms are responsible for the observed variability and that instability of the chemical feedback loops plays a role in the resulting breathing pattern [18, 19].

During anesthesia and procedural sedation, various drugs with potential effects on the regulation of breathing are administered. For instance, it is acknowledged that midazolam can impair MV mainly through a decrease in TV and to a lesser extent through a decrease in RR [20]. In contrast, s-ketamine is known to activate breathing with an increase in both RR and inspiratory time [6–8, 21, 22]. The changes in respiratory variability after administering s-ketamine, midazolam, or procedural sedation in general, are unknown.

In this study, we investigated the effects of a small single dose of midazolam or s-ketamine on RR, TV, and MV as well as their variability in patients undergoing procedural sedation with propofol and remifentanil. We hypothesized that respiratory variability is impaired more after administration of midazolam than after administration of s-ketamine. To test our hypothesis we studied respiratory variables as well as their variability with a non-invasive impedance-based respiratory volume monitor as described previously [17]. We indeed observed marked differences between midazolam and s-ketamine.

## Methods

### Study population

This prospective, observational, single-center cohort study was approved by the research ethics committee of Amsterdam UMC (location VUmc, 2019.558, 16 October 2019). We included 20 patients undergoing procedural sedation and analgesia (PSA) for pulmonary vein isolation for drug-refractory paroxysmal atrial fibrillation, between October 2019 and June 2020. Written informed patient consent was obtained from all participants. Patients younger than 18 years of age at the time of inclusion or with an allergy for adhesives were excluded.

### Sedation and analgesia

All patients were seen at the pre-operative clinic. PSA was conducted in accordance with the Dutch guidelines and local protocols for the administration of PSA outside the operating room. All patients received propofol (Propofol-Lipuro, Braun, Germany, 50–142 µg/kg/min) and remifentanil (Mylan B.V., The Netherlands, 13–44 ng/kg/min) via target-controlled infusion. In addition, patients received a single dose of either midazolam (Aurobindo Pharma B.V., The Netherlands, 1–3 mg) or s-ketamine (Ketanest-S, Eurocept Pharmaceuticals, The Netherlands, 10–25 mg) simultaneously with initiation of the target-controlled infusion of propofol and remifentanil. Both drugs are included in our local protocols as additional drugs to be used during PSA. The dosage of propofol, remifentanil and midazolam or s-ketamine was titrated to the desired clinical effect by a certified registered nurse anesthetist specialized in PSA with a supervising anesthetist. Supplemental oxygen was administered when necessary to maintain SpO_2_ > 94%.

### Pulmonary vein isolation

After induction of PSA and after the single dose of midazolam or s-ketamine, the cardiologist accessed the femoral vein with a Seldinger technique and introduced a trans-septal catheter into the left atrium. Focal catheter ablation with radiofrequency current was applied to achieve pulmonary vein isolation. A 3-D electro-anatomical mapping system was used to navigate this catheter [23].

### Standard monitoring

Pulse oximetry (SpO_2_), heart rate, electrocardiography and capnography were monitored continuously while non-invasive blood pressure was measured intermittently (IntelliVue MX450, M3015B Phillips NV, The Netherlands).

### Measurement of respiratory variables

Respiratory variables (respiratory rate [RR], changes in tidal volume [TV], and changes in minute ventilation [MV]) were continuously measured before, during and after PSA using an impedance-based superficial respiratory volume monitor (ExSpiron, Respiratory Motion, Walthan, MA, US) with a thoracic electrode [17, 24]. Respiratory measurements were commenced at least 30 min before induction of PSA and were continued until discharge from the post-anesthesia care unit. This involved a 30 s baseline measurement of TV and MV during a period of normal breathing (i.e. without any anesthetic), after which changes in TV and MV were recorded as “% of baseline”. Data (RR, TV, MV) were recorded by the respiratory volume monitor as average values during intervals of 1 min. Data were transferred by an encrypted USB memory stick to a secured desktop computer for further analysis.

### Statistical analysis

This study was performed in accordance with the strengthening the reporting of observational studies in epidemiology (STROBE) guidelines [25].

For sample size calculation, variability of MV was defined as the primary outcome. It was calculated that 10 subjects are required per group in order to detect a 25% difference in variability between the midazolam group and the s-ketamine group; the α value was 0.05 and the power (1 – β) was 0.80. Data were analyzed using SPSS (Version 22.0, IBM, Armonk, NY) and R (2017, R Core Team, Vienna, Austria). Normally distributed data are presented as mean ± standard deviation (SD) and non-parametric data as median with interquartile range (IQR). Variability over time of RR, TV and MV were calculated as the coefficient of variation, defined as the ratio of the SD and the mean. Variability of RR, TV and MV were calculated before PSA, during PSA and after PSA, over time periods lasting 30 min each.

Differences between groups were analyzed using the unpaired Student’s t-test. Changes within groups were analyzed with the paired Student’s t-test. Significance was assumed with a two-sided p-value of < 0.05.

## Results

We included 20 patients undergoing PSA with propofol and remifentanil. Ten patients received an additional administration of midazolam while the other 10 received an additional administration of s-ketamine. Demographics of the two groups are given in Table 1. There were no significant differences in age, gender, body mass index, American Society of Anesthesiologists classification, pre-procedural SpO_2_, duration of sedation or dosage of other drugs between the two groups (with exception of midazolam or s-ketamine), respectively.

**Table 1:** Patient characteristics

	Midazolam (n = 10)	s-ketamine (n = 10)
Age; years	62 (10)	56 (7.2)
Males	7	8
Body Mass Index; kg m^−2^	26 (2.8)	27 (4.5)
ASA classification		
2	8	6
3	2	4
Pre-procedural SpO2; %	98 (1.4)	98 (1.7)
COPD	0	1
Duration of sedation; mins	159 (42.5)	170 (31.4)
Propofol infusion (total); mg	989 (390)	1117 (383)
Propofol infusion; mg kg^−1^ h^−1^	4.58 (0.89)	4.72 (1.68)
Remifentanil infusion (total); µg	313 (97)	354 (115)
Remifentanil infusion; µg kg^−1^ h^−1^	1.51 (0.48)	1.51 (0.53)
Ketamine dose; mg	–	20 [12–20
Midazolam dose; mg	1.0 [1.0–2.0]	–

*ASA* American Society of Anesthesiologists; *COPD* chronic obstructive pulmonary disease

Data are shown as mean (SD), median [IQR] or frequency

**Fig. 1:**
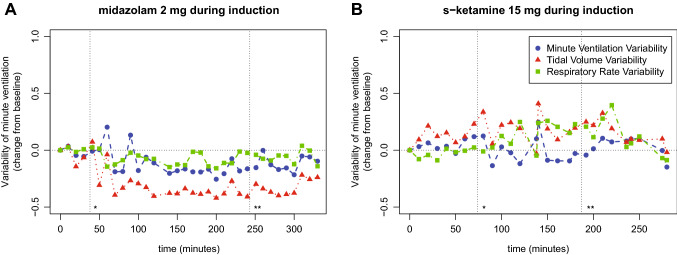
A representative registration of the variability of minute ventilation, tidal volume an respiratory rate before, during and after procedural sedation with propofol and remifentanil. One patient **A** received an additional dose of midazolam during induction, another patient **B** received an additional dose of s-ketamine during induction. *Start of procedural sedation, **end of procedural sedation

A representative registration of the respiratory variability of one patient from the midazolam group and one patient from the s-ketamine group is shown in Fig. 1.

**Fig. 2:**
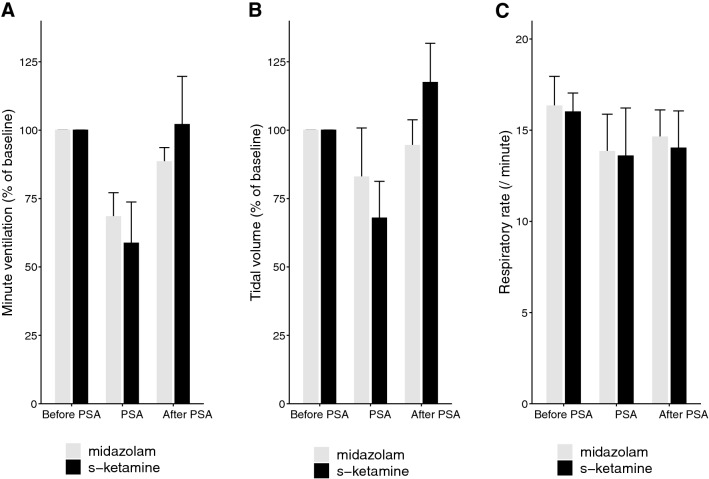
Minute ventilation (**A**), tidal volume (**B**), and respiratory rate (**C**) before, during and after procedural sedation with propofol and remifentanil (n = 20). While one group received an additional dose of midazolam (n = 10, grey bars), the other group received an additional dose of s-ketamine (n = 10, black bars). There were no differences between the midazolam and s-ketamine group. Measurements of minute ventilation and tidal volume are expressed as % of baseline. *PSA* procedural sedation and analgesia

In both groups, we observed a decrease in TV and MV during PSA when compared to baseline. The mean decrease of MV was – 31.4% ± 13.9% (p < 0.0001) in the midazolam group, and – 41.1% ± 24.4% (p = 0.002) in the s-ketamine group. The mean decrease of TV was – 16.9% ± 28.6% (p = 0.07) in the midazolam group, and – 32.0% ± 21.4% in the s-ketamine group (p=0.003). There was no significant change in RR during PSA; mean difference – 2.5 ± 3.9 breaths/min (p = 0.073) in the midazolam group, and – 2.4 ± 3.9 breaths/min (p = 0.084) in the s-ketamine group. All three variables increased again after termination of PSA and almost returned to baseline within one hour. There was no significant difference in RR, TV and MV between patients receiving midazolam or s-ketamine, see Fig. 2.

**Fig. 3:**
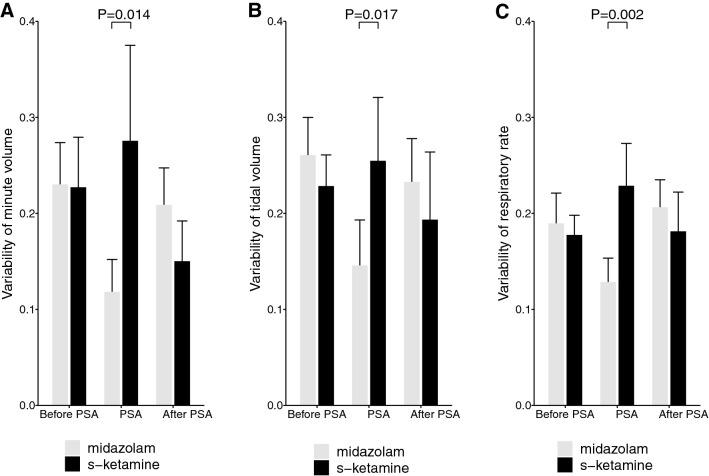
Variability of minute ventilation (**A**), tidal volume (**B**), and respiratory rate (**C**) before, during and after procedural sedation with propofol and remifentanil (n = 20). While one group received an additional dose of midazolam (n = 10, grey bars), the other group received an additional dose of s-ketamine (n = 10, black bars). In the midazolam group variability of all three respiratory variables was significantly decreased while in the variability in the s-ketamine group remained unchanged. *PSA* procedural sedation and analgesia

In contrast, we observed a significant difference between both groups with regard to the variability of the three respiratory variables. In the group receiving midazolam, the variability of all variables significantly decreased during PSA when compared to baseline; MV 0.23 ± 0.07 vs. 0.11 ± 0.05 (p = 0.011), TV 0.26 ± 0.06 vs. 0.14 ± 0.08 (p = 0.003), RR 0.19 ± 0.05 vs. 0.13 ± 0.04 (p = 0.011). Variability remained unchanged in the group receiving s-ketamine; MV 0.23 ± 0.08 vs. 0.27 ± 0.16 (p = 0.182), TV 0.23 ± 0.05 vs. 0.25 ± 0.11 (p = 0.390), RR 0.18 ± 0.03 vs. 0.23 ± 0.07 (p = 0.129). Variability of MV, TV, and RR during PSA was significantly lower in the group receiving midazolam than in the group receiving s-ketamine (p = 0.014, p = 0.017, p = 0.002, respectively), see Fig. 3.

## Discussion

In spontaneously breathing patients undergoing procedural sedation with propofol and remifentanil, the variability of RR, TV, and MV was significantly reduced by a single dose of midazolam whereas they remained unchanged when patients received s-ketamine, respectively. These observations suggest that midazolam and s-ketamine differ in their effects on spontaneous respiration.

### Critique of methods

For the interpretation and extrapolation of our results some methodological aspects should be considered. All measurements were performed in spontaneously breathing patients before, during and after PSA with propofol and remifentanil. While one group received a single dose of midazolam, the other received a single dose of s-ketamine in a non-randomized fashion. Also, midazolam or s-ketamine was administered concomitantly with induction of PSA. Thus, although unlikely, we cannot exclude that other results would have been recorded in patients receiving only midazolam or s-ketamine, without propofol and remifentanil. Second, we administered a single small dose of midazolam or s-ketamine and we cannot extrapolate our conclusions to higher or repeated doses. Further studies are required to establish the dose-response relationship. Third, a non-invasive impedance-based technique was used to measure respiratory volume variables before, during and after PSA. Previous studies reported acceptable accuracy of this technique [14, 26–28]. Nevertheless, measurements may be influenced by various procedures such as the use of radio-frequent energy [23]. To exclude any possible interferences all measurements were completed before interventions were started.

### Interpretation of results

In the present study, we hypothesized that midazolam and s-ketamine have different effects on the variability of respiratory variables during PSA. Consistent with this hypothesis, we observed marked differences between the midazolam and the s-ketamine group. While the variability significantly decreased with midazolam, no changes were observed with s-ketamine. Our observations may be explained by the different pharmacological pathways of midazolam and s-ketamine. While benzodiazepines alter the control of breathing through gamma-amino butyric acid (GABA)_A_ and glycine-mediated pathways [1, 5], s-ketamine induces a sympathico-adrenal activation [22]. It is acknowledged that GABA is essential for the generation of respiratory rhythm and is responsible for tonic inhibition of both inspiratory and expiratory neurons, which would suggest a major role in the regulation of the variability of breathing [4, 29]. In contrast, s-ketamine does not affect GABA-receptors but stimulates respiration indirectly through arousal [21].

It is likely that respiratory variability has physiological functions also during anesthesia and procedural sedation. For example, episodes of deep breathing may recruit dependent alveolar regions that otherwise would collapse. High respiratory variability is associated with lower organ failure score in the intensive care unit [16], increased successful extubation [17], lower asthma severity in children [12], and less re-hospitalizations due to respiratory disease in neonates [15]. S-ketamine but not midazolam preserves respiratory variability and may perhaps be more suitable for patients at risk of respiratory complications. A larger, randomized controlled trial is necessary to confirm these findings.

In conclusion, the variability of breathing is reduced by midazolam but not by s-ketamine. We suggest that midazolam and s-ketamine affect regulation of breathing in a different way.
